# Fine Morphology of the Proboscis and Associated Sensilla in *Pontia edusa* (Fabricius, 1777) (Lepidoptera: Pieridae)

**DOI:** 10.3390/insects17040392

**Published:** 2026-04-03

**Authors:** Ya-Rong Gu, Jia-Qi Yuan, Chao Gao, Ying Miao

**Affiliations:** 1 School of Agriculture, Ningxia University, Yinchuan 750021, China; 12023131408@stu.nxu.edu.cn; 2 College of Plant Protection, Hebei Agricultural University, Baoding 071001, China; 18161778820@163.com; 3 College of Plant Protection, Anhui Agricultural University, Hefei 230036, China; gaoc@ahau.edu.cn

**Keywords:** Pieridae, proboscis zonation, dorsal legulae, sensilla, SEM

## Abstract

*Pontia edusa* is a nectar-feeding butterfly widely distributed in Europe, northwestern India, Siberia, North Africa (extending to Ethiopia), and most regions of China; however, detailed information on the fine morphology of its proboscis remains limited. In this study, scanning electron microscopy was used to examine the external morphology, zonal organization, and sensilla composition of the adult proboscis, and sexual differences in selected morphological parameters were statistically analyzed. The proboscis consists of a pair of elongated maxillary galeae that interlock via dorsal and ventral legulae to form a continuous food canal. Based on structural variation in the dorsal legulae, the proboscis can be divided into three zones. Zone 1 is characterized by tightly interlocked legulae and serrated external ridges; Zone 2 shows enlarged interlegular gaps and gradual modification of external surface ornamentation; and Zone 3 lacks dorsal legulae and bears flake-like bumps. Three major types of sensilla were identified on the proboscis, including sensilla chaetica, basiconica, and styloconica, comprising six subtypes in total. The sensilla chaetica subtype sc2, restricted to the ventral surface of the proximal proboscis, represents a previously unreported morphological feature for *P. edusa*. These results provide a detailed morphological framework for the proboscis of *P. edusa* and expand the comparative basis for evaluating structural diversity and regionalization patterns of the proboscis in flower-visiting pierid butterflies.

## Simple Summary

This study investigates the external morphology, zonal organization, and sensilla composition of the proboscis in adult *Pontia edusa* using scanning electron microscopy. The proboscis is slender and shows no marked sexual difference in overall morphology. Based on structural variation in the dorsal legulae, the proboscis is subdivided into three distinct zones. Three major sensillum types with six subtypes were identified: sensilla chaetica (sc1, sc2), sensilla basiconica (sb1–sb3), and sensilla styloconica (ss). These sensilla differ in morphology and spatial distribution along the proboscis. The study provides a detailed morphological description of the proboscis of *P. edusa* and contributes comparative data for future investigations of proboscis diversity and structural evolution within Pieridae.

## 1. Introduction

The proboscis is a defining feeding organ of adult Lepidoptera within Glossata and is formed by the interlocking of two elongated maxillary galeae. These galeae are connected by dorsal and ventral legulae, and their opposing concave inner surfaces together enclose the food canal [1,2,3]. As the central structure underlying adult feeding behavior, the proboscis has attracted sustained morphological interest and has been investigated in a wide range of lepidopteran taxa, including Noctuidae [4,5,6,7,8], Pyralidae [9], Sphingidae [10], Hesperiidae [11], Nymphalidae [12,13,14,15], and Papilionidae [12]. Various types of sensilla distributed along the proboscis play crucial roles in insect foraging, host recognition, and oviposition site selection. Six major types of sensilla have been identified on lepidopteran proboscides based on external morphology, including sensilla chaetica, basiconica, styloconica, coeloconica, filiformia, and campaniformia [2]. Among these, sensilla chaetica, basiconica, and styloconica are relatively common [13,16,17]. Accumulating evidence indicates that variation in proboscis morphology is closely linked to adult feeding modes and resource exploitation strategies [18,19,20].

Lehnert et al. [12] proposed a three-zone model of the proboscis in flower-visiting Lepidoptera, in which Zone 1 extends from the head-proboscis junction to the point where the upper branch of the dorsal legulae begins to expand, Zone 2 spans from this expansion region to the point where the dorsal legulae disappear, and Zone 3 comprises the distal region lacking dorsal legulae. Zone 3 has been documented in some flower-visiting species, such as *Papilio glaucus* (Papilionidae), *Danaus Plexippus* (Danaidae), *Pieris rapae* (Pieridae)*, Scotogramma trifolii*, *Protoschinia scutosa* and *Helicoverpa armigera* (Noctuidae) [12,21,22], but absent in others [5,12,22], indicating that the presence and boundaries of Zone 3 are taxonomically variable and remain unresolved.

Within Pieridae, species of *Pieris* and *Colias* possess a distinct Zone 3 [12,23,24,25]; however, the occurrence and stability of this region in other pierid genera, including *Pontia*, have not been systematically evaluated. *Pontia edusa* is a widespread flower-visiting species common in Europe, northwestern India, Siberia, North Africa, Ethiopia, and most regions of China [26,27,28]. Although Mengi and Çalişkan [29] described the proboscis morphology and sensillum types of this species, detailed information on its proboscis zonal organization and distribution of sensilla on the proboscis is lacking.

In the present study, scanning electron microscopy (SEM) was used to investigate the fine morphology of the adult proboscis and its associated sensilla in *P. edusa*. The aims of this work were (1) to characterize the zonal organization of the proboscis in this species, with particular emphasis on the occurrence and boundaries of Zone 3, and (2) to document the distribution and diversity of proboscis sensilla. By generating these data, this study seeks to supplement comparative data on proboscis regionalization within Pieridae and to contribute new evidence for evaluating the taxonomic and evolutionary stability of proboscis structures in flower-visiting butterflies.

## 2. Materials and Methods

### 2.1. Specimen Collection

Adult *P. edusa* were collected in mid-July 2023 and 2024 from Ningxia Hui Autonomous Region, China (38°30′10″ N, 106°8′20″ E). Specimens used in this study were assigned to *P. edusa* based on geographic distribution, diagnostic genitalia morphology, and *cox1* sequence data, which distinguish them from the closely related *P. daplidice*. Only intact and healthy individuals were selected for subsequent morphological observation and quantitative analyses.

### 2.2. Dissection and Fixation

Live adults were anesthetized with ether (Sinopharm Chemical Reagent Co., Ltd., Shanghai, China) prior to dissection. The proboscis was carefully excised under a stereomicroscope (SMZ745T, Nikon Corporation, Tokyo, Japan). Immediately after dissection, proboscides were fixed in 0.1 mol/L phosphate buffer (pH 7.2, prepared by mixing 36 mL 0.2 mol/L Na_2_HPO_4_, 14 mL 0.2 mol/L NaH_2_PO_4_ (Tianjin Damao Chemical Reagent Factory, Tianjin, China) and 50 mL distilled water) containing 2.5% glutaraldehyde (Sinopharm Chemical Reagent Co., Ltd., Shanghai, China) at 4 °C for 24 h to preserve tissue morphology. The remaining specimens were preserved in 75% ethanol (Shandong Lircon Medical Technology Co., Ltd., Dezhou, China) and deposited in the Entomological Collection of the School of Agriculture, Ningxia University, Yinchuan, China (NXU).

### 2.3. SEM Preparation and Observation

Fixed specimens were dehydrated through a graded ethanol series (30%, 50%, and 70% for 10 min each; 80% for 15 min; 90% for 20 min; and 100% for 30 min, repeated twice). Samples were subsequently transferred through a graded ethanol–tert-butanol series (3:1, 1:1, and 1:3; 30 min each), followed by immersion in pure tert-butanol (Shandong Lircon Medical Technology Co., Ltd., Dezhou, China) for 30 min. After freeze-drying for 3 h, samples were mounted on stubs, sputter-coated with gold, and examined using a scanning electron microscope (S-3400N, Hitachi High-Technologies Corporation, Tokyo, Japan) at an accelerating voltage of 15 kV.

### 2.4. Morphological Measurements

The total length of the proboscis and morphological parameters of each proboscis zone were quantified using a stereomicroscope and SEM images. Measurements were obtained from 45 adults (20 females and 25 males), following the protocol described by Kramer et al. [15].

For whole-proboscis measurements, freshly dissected proboscides were gently uncoiled, fixed onto foam boards with insect pins, and photographed at 75× *g* magnification using a stereomicroscope (Leica M205 A, Wetzlar, Germany) equipped with a CCD camera. Linear measurements were performed using the segmented line tool in ImageJ 1.50i (National Institutes of Health, Bethesda, MD, USA) software [30]. Based on morphological variation in the dorsal legulae, the proboscis was subdivided into three zones. The width of Zones 1 and 2 was measured at 200× *g* magnification. The length and width of the upper branch of the dorsal legulae were measured at 750× *g* magnification, with three legulae measured per individual in each zone. Measurements of sensilla were conducted on SEM images at 1000× *g* or 15,000× *g* magnification, depending on sensillum size and surface complexity. For each sensillum type in each zone, five sensilla were randomly selected and measured for length and basal width.

To avoid pseudo-replication, measurements from multiple legulae or sensilla within the same individual were averaged to obtain a single mean value per individual prior to statistical analysis.

### 2.5. Statistical Analysis

All statistical analyses were performed using SPSS v20.0 (SPSS Inc., Chicago, IL, USA). Data are presented as mean ± standard deviation (SD). Differences between males and females were assessed using independent-samples *t*-tests, with a significance level set at *p* < 0.05.

### 2.6. Terminology

Terminology for proboscis structures was adopted from Lehnert et al. [12]. Classification and nomenclature of sensilla followed Faucheux [2].

## 3. Results

### 3.1. Morphological Characteristics of the Proboscis

**Figure 1:**
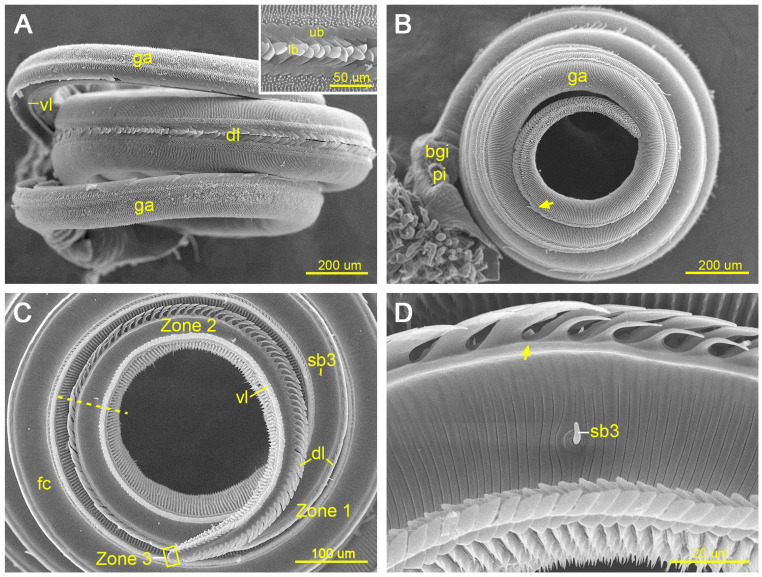
Proboscis of *Pontia edusa*. (**A**) Dorsal view of the proboscis; the inset shows detailed structure of the upper and lower branches of the dorsal legula. (**B**) Lateral view of the proboscis; the arrow indicates the boundary between Zones 1 and 2. (**C**) Inner view of galea, showing the three zones (Zones 1–3) and the dorsal and ventral legulae. (**D**) Close-up of the dotted line of (**C**), showing the transition from Zone 1 to 2, where the lower branch of the dorsal legula is markedly reduced. The dotted line and arrows indicate the boundary between Zones 1 and 2, and the rectangle indicates Zone 3. bgi, basal proboscis joint; dl, dorsal legula; fc, food canal; ga, galea; lb, lower branch of the dorsal legula; pi, pilifer; sb3, sensilla basiconica subtype 3; ub, upper branch of the dorsal legula; vl, ventral legula.

In adult *P. edusa*, the proboscis is formed by two elongated maxillary galeae that interlock via dorsal and ventral legulae to enclose the food canal (Figure 1A). At rest, the proboscis is coiled into four to five spiral turns and articulates proximally with the head capsule. In the lateral view, the proboscis gradually tapers from the basal region toward the distal tip (Figure 1B).

No significant sexual difference was detected in the total proboscis length between females (11,193.26 ± 931.10 μm, *n* = 20) and males (10,706.71 ± 612.87 μm, *n* = 24; *p* > 0.05; Table 1). Based on consistent changes in the morphology of the dorsal legulae, the proboscis can be subdivided into three distinct zones (Zones 1–3; Figure 1C). Zone 1 extends from the proboscis base to the region where the lower branch of the dorsal legulae becomes distinctly shortened and narrower than the upper branch. Zone 2 extends from this transition region, in which the enlarged upper branch clearly overlaps the reduced lower branch, to the point where the dorsal legulae disappear. Zone 3 represents the distalmost zone, where dorsal legulae are completely absent.

#### 3.1.1. Zone 1

Zone 1 occupies more than 90% of the total proboscis length. The length of Zone 1 differed significantly between females (10,895.60 ± 953.08 μm, *n* = 13) and males (10,000.44 ± 749.85 μm, *n* = 21; *p* < 0.05), whereas no significant sexual difference was observed in zone width (Table 1).

**Figure 2:**
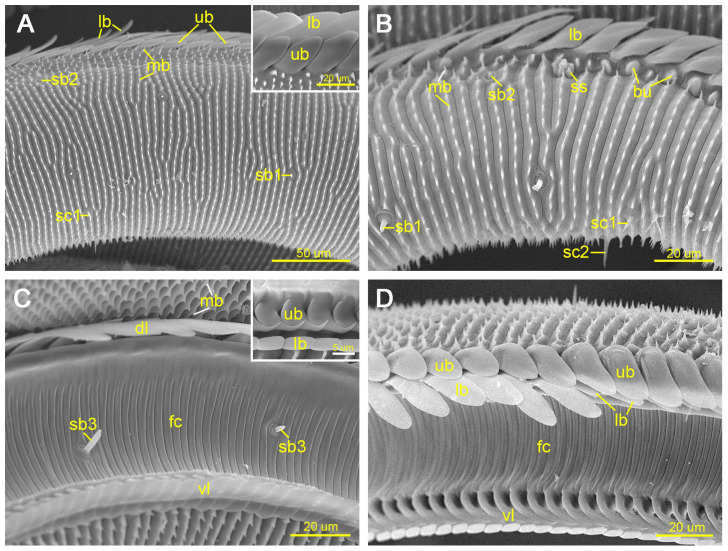
Zone 1 of the proboscis. (**A**) Proximal external surface of Zone 1; the inset shows the upper and lower branches of the dorsal legula. (**B**) Distal external surface of Zone 1, showing the types of sensilla distributed on the external surface of the proboscis. (**C**) Proximal internal surface of Zone 1 (food canal); the inset shows the upper and lower branches of the ventral legula. (**D**) Distal internal surface of Zone 1 (food canal), illustrating the transition in the lower branch of the dorsal legula from Zone 1 to Zone 2. bu, bump; dl, dorsal legula; fc, food canal; lb, lower branch of dorsal or ventral legula; mb, microbump; sb1/sb2/sb3, sensilla basiconica 1/2/3; sc1/sc2, sensilla chaetica 1/2; ss, sensilla styloconica; ub, upper branch of dorsal or ventral legula; vl, ventral legula.

The external surface of the galeae in Zone 1 is densely covered with transverse ridges bearing serrated microbumps (Figure 2A,B). In contrast, the internal surface forming the food canal is smooth and characterized by evenly spaced transverse grooves (Figure 2C,D). Each dorsal legula consists of an upper and a lower branch. The upper branch is rectangular near the proboscis base and gradually becomes lanceolate toward the distal region (Figure 2A,D). The lower branch forms a smooth, willow-leaf-shaped flattened lamella that overlaps longitudinally with the upper branch, creating narrow interlocking gaps (Figure 2A). Ventral legulae comprise a hook-shaped upper branch and an elongated lower branch (Figure 2C).

The length of the upper branch of the dorsal legulae in Zone 1 showed a highly significant sexual difference (*p* < 0.01), whereas its width did not differ significantly between females and males (Table 1).

#### 3.1.2. Zone 2

The length of Zone 2 differed significantly between females (583.53 ± 61.38 μm, *n* = 13) and males (644.88 ± 64.38 μm, *n* = 21; *p* = 0.01), whereas no significant difference was detected in zone width (Table 1).

**Figure 3:**
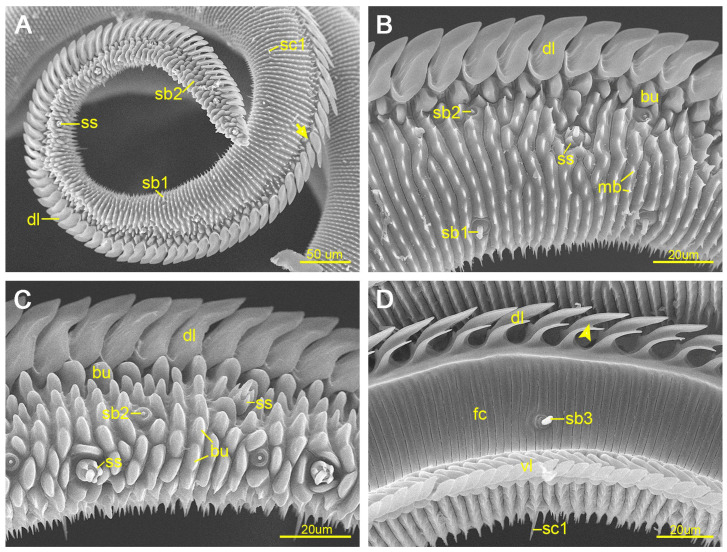
Zone 2 of the proboscis. (**A**) External view of the proboscis, showing the arrangement of the dorsal legulae; the arrow indicates the boundary between Zones 1 and 2. (**B**) Proximal external surface of Zone 2, showing a sensillum styloconica (ss). (**C**) Distal external surface of Zone 2, showing numerous blunt protrusions. (**D**) Internal surface of the middle segment of Zone 2; the arrow indicates that the dorsal legula has a central groove. bu, bump; dl, dorsal legula; fc, food canal; mb, microbulge; sb1/sb2/sb3, sensilla basiconica 1/2/3; sc1, sensilla chaetica 1; ss, sensilla styloconica; vl, ventral legula.

The proximal external surface of Zone 2 is covered with serrated transverse ridges similar to those observed in Zone 1 (Figure 3A,B), whereas the distal external surface bears numerous blunt bumps of variable shapes and sizes (Figure 3A,C). The internal surface morphology of Zone 2 resembles that of Zone 1. In this zone, the upper branch of each dorsal legulae elongates further and completely overlaps the shortened lower branch. Each dorsal legula is inclined toward the proboscis tip and possesses a distinct central groove (Figure 3D). The ventral legulae retain a morphology similar to that observed in Zone 1.

Significant sexual differences were detected in both the length (*p* < 0.05) and width (*p* < 0.01) of the upper branch of the dorsal legulae in Zone 2 (Table 1).

#### 3.1.3. Zone 3

**Figure 4:**
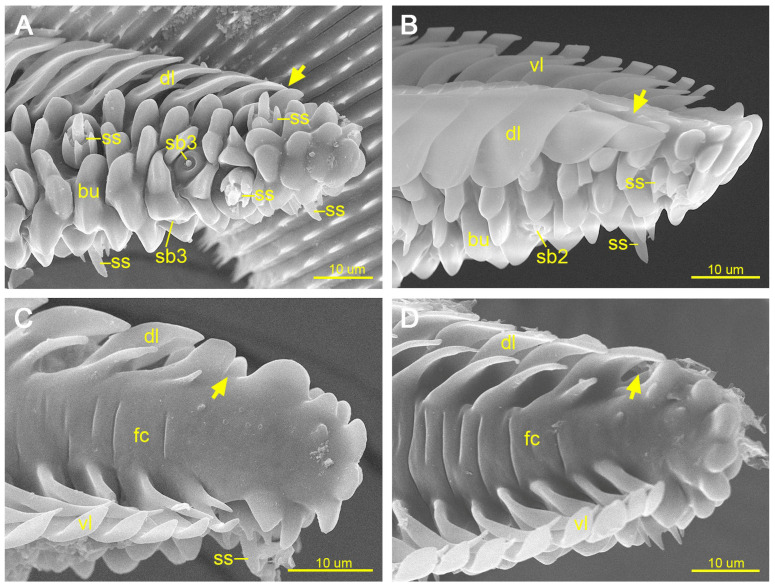
Zone 3 of the proboscis. (**A**) Lateral view of Zone 3, showing numerous sensilla styloconica and flake-like bumps. (**B**) Dorsal view of Zone 3; showing the connection between the dorsal and ventral legulae. (**C**) Inner surface of Zone 3 in a female. (**D**) Inner surface of Zone 3 in a male. The transverse grooves present on the internal surface in Zones 1 and 2 are absent in Zone 3. Arrows indicate the boundary between Zones 2 and 3. bu, bump; dl, dorsal legula; fc, food canal; sb2/sb3, sensilla basiconica 2/3; ss, sensilla styloconica; vl. ventral legula.

The external surface is densely covered with flake-like bumps (Figure 4A), and the dorsal and ventral legulae gradually merge (Figure 4B). On the internal surface, transverse grooves become progressively more widely spaced toward the distal end of Zone 2 and are completely absent in Zone 3. Minor sexual variation in the internal surface morphology of Zone 3 was observed between females and males (Figure 4C,D). The ventral legulae exhibit a morphology comparable to that observed in Zones 1 and 2.

### 3.2. Morphology and Distribution of Proboscis Sensilla

A total of three major sensillum types and six subtypes were identified on the proboscis of *P. edusa*. These include two subtypes of sensilla chaetica (sc1 and sc2), three subtypes of sensilla basiconica (sb1, sb2, and sb3), and sensilla styloconica (ss).

#### 3.2.1. Sensilla Chaetica

**Figure 5:**
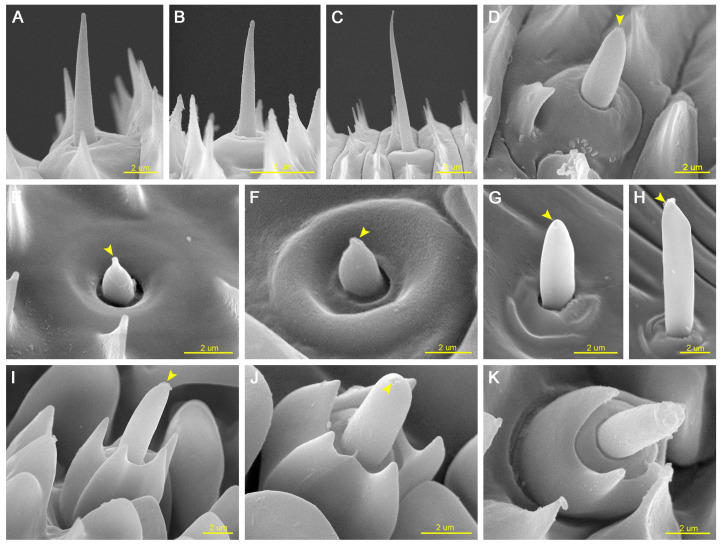
Sensilla on the proboscis of *Pontia edusa*. (**A**) Sensilla chaetica 1 (sc1) in Zone 1. (**B**) Sensilla chaetica 1 (sc1) in Zone 2. (**C**) Sensilla chaetica 2 (sc2) in Zone 1. (**D**) Sensilla basiconica 1 (sb1). (**E**) Sensilla basiconica 2 (sb2). (**F**) Sensilla basiconica 2 (sb2), surrounded by annular cuticular protrusions. (**G**) Sensilla basiconica 3 (sb3, short type) in the food canal. (**H**) Sensilla basiconica 3 (sb3, long type) in the food canal. (**I**) Sensilla styloconica (ss), with the stalk (st) bearing five longitudinal ridges. (**J**) Sensilla styloconica (ss), showing a single-porous sensory cone. (**K**) Sensilla styloconica (ss), with the stalk (st) bearing four longitudinal ridges. Arrows indicate terminal pores.

Sensilla chaetica are elongated, non-porous, bristle-like sensilla that gradually taper from base to tip and are inserted into circular sockets. Two subtypes were identified. Sensilla chaetica 1 (sc1) are short bristles distributed on the external surface of Zones 1 and 2 (Figure 5A,B). Sensilla chaetica 2 (sc2) are longer bristles (Figure 5C) exclusively distributed on the ventral surface of Zone 1 (Figure 2B).

No significant sexual difference was detected in the length of sc1, whereas the basal width differed significantly between females and males (*p* < 0.05). Neither the length nor the basal width of sc2 showed significant sexual differences (Table 1).

#### 3.2.2. Sensilla Basiconica

Sensilla basiconica are single-porous, blunt-tipped conical sensilla inserted into circular sockets. They are distributed on both the external surface and the internal surface (food canal) of the proboscis in Zones 1 and 2. Based on morphology and distribution, three subtypes were identified. Sensilla basiconica 1 (sb1) are long cones (Figure 5D), whereas sensilla basiconica 2 (sb2) are short cones (Figure 5E,F); both are distributed on the external surface of the proboscis. Sensilla basiconica 3 (sb3) are restricted to the internal surface of the proboscis and are arranged in a single longitudinal row extending from Zone 1 to the distal two-thirds of Zone 2. The cones of sb3 exhibit noticeable variation in length (Figure 5G,H).

With the exception of the basal width of sb1, which differed significantly between sexes (*p* < 0.05), no significant sexual differences were detected in the remaining measured parameters of sensilla basiconica (Table 1).

#### 3.2.3. Sensilla Styloconica

Sensilla styloconica (ss) are sparsely distributed on the external surface of Zones 2 and 3 and become progressively more concentrated toward the distal tip of the proboscis. Each ss consists of a single-porous cone surrounded by a star-shaped stylus bearing four to five short longitudinal ridges. The ridges vary in prominence, and the apical protrusion is short and blunt (Figure 5I–K).

Both the length and basal width of ss differed significantly between females and males (*p* < 0.05; Table 1). In addition, the stylus length exhibited a significant sexual difference between females (6.49 ± 0.46 μm, *n* = 9) and males (6.11 ± 0.36 μm, *n* = 12; *p* < 0.05), whereas no significant differences were detected in the length or width of the cone (Table 1).

## 4. Discussion

The proboscis of *Pontia edusa* is slender, coiled, and gradually tapers from the basal region toward the distal tip. The galeae exhibit a rough external surface and a smooth internal surface, with dorsal and ventral legulae distributed along their length, and three major sensillum types present on the proboscis. These general characteristics are consistent with the proboscis morphology described for most flower-visiting Lepidoptera and the descriptions of this species by Mengi and Çalişkan [4,5,6,9,10,13].

The interlocking of the dorsal and ventral legulae represents a fundamental structural feature of the lepidopteran proboscis [2,12,31]. In *P. edusa*, as in other Lepidoptera [16,32], the legulae are composed of upper and lower branches, and the legulae of opposing galea overlap to enclose the food canal. The ventral legulae of *P. edusa* show a relatively uniform morphology along the proboscis, consisting of a hook-shaped upper branch and an elongated lower branch. This configuration closely resembles that reported in other flower-visiting species, where hook-shaped lamellae interlock in a zipper-like manner [33].

In contrast, the dorsal legulae of *P. edusa* exhibit pronounced morphological variation along the length of the proboscis. On the basis of these changes, the proboscis can be subdivided into three zones, consistent with the regionalization pattern proposed by Lehnert et al. [12]. In Zone 1, the upper branch of the dorsal legulae is relatively small, whereas the lower branch is well developed, and the lower branches from opposing galeae overlap to form a narrow interlegular gap. This structural configuration is comparable to that observed in the basal regions of the proboscis in other pierid butterflies [12,23].

In Zone 2, each dorsal legula bears a distinct central groove, a feature repeatedly reported in flower-visiting Lepidoptera [2,12,13,20,22] and generally interpreted as being associated with liquid uptake processes [5]. In Zone 3 of *P. edusa*, the dorsal legulae are completely absent. This feature is consistent with observations in *Pieris* and *Colias* and appears to be a stable morphological trait within Pieridae [12,23,24,25].

The external surfaces of Zones 1 and 2 are densely ornamented with transverse ridges bearing serrated microbumps, which gradually transform into blunt bumps toward the distal proboscis. Similar ornamentation occurs in *Pieris rapae*, *Colias erate* and *Aporia crataegi* [12,23,24], and resembles surface patterns reported in Noctuidae [4,5,6], Pyralidae [9], and Sphingidae [10]. In contrast, proboscis surface structures in Hesperiidae are more diverse, including rounded, tile-like, and oblique ridge-shaped protrusions [11,34]. In Nymphalidae, the external surface typically consists of rows of rounded microbumps that progressively develop serrated margins [11,12,13,14], whereas in Papilionidae, rounded microbumps extend toward regions adjacent to the dorsal legulae [12]. Although such surface features have often been discussed in relation to proboscis maintenance and resistance to contamination [1,19,35], their functional and evolutionary significance remains insufficiently explored and warrants further comparative investigation across a broader taxonomic range.

Three major sensillum types, including sensilla chaetica, basiconica, and styloconica, were identified on the proboscis of *P. edusa*, consistent with the sensillum composition previously reported by Mengi and Çalişkan [29]. Sensilla chaetica, distributed across the entire outer surface of the proboscis, are especially long in the proximal region and likely participate in mechanical sensing and nutrient localization [1,13]. Sensilla basiconica occur externally and internally, both bearing peg-like sensory cones. External sensilla basiconica are arranged in a single row along the entire galeal surface, whereas internal sensilla basiconica occur in smaller numbers on the inner wall of the food canal and may function in detecting the flow rate or composition of ingested liquid [13]. In addition, the present study newly documents a second subtype of sensilla chaetica 2 (sc2), which is restricted to the ventral surface of Zone 1 and is markedly longer than sensilla chaetica 1 (sc1). Similar ventrally distributed sensilla have been reported in *Vanessa cardui*, *Melitaea cinxia* (Nymphalidae), and *Zerynthia Polyxena* (Papilionidae), and may contact adjacent coils when the proboscis is coiled at rest, potentially providing tactile feedback related to proboscis positioning [13,35,36,37]. The appearance of sc2 in our specimens, but not in those examined by Mengi and Çalırkan [38], may reflect geographic variation between Turkish and Chinese populations, or other interpopulation differences that warrant further investigation.

Sensilla styloconica are widely regarded as having evolved from sensilla basiconica, with modification of the socket into a stylus-like structure [2,37]. The morphology of the stylus varies considerably among lepidopteran taxa and has been proposed as a character of potential taxonomic value [2,16,25]. Stylus morphology has been categorized into smooth cylindrical, spherical, and star-shaped forms bearing longitudinal ridges [25]. In Pieridae, sensilla styloconica typically possess star-shaped styli, but distinct intergeneric differences are evident. In *Pieris*, the stylus is smooth, approximately equal in length to the cone, bears three to five inconspicuous ridges, and has a sharp apical protrusion [12]. In *Aporia*, the stylus is smooth, shorter than the cone, bears four weak ridges, and has a blunt apical protrusion [24]. In *Colias*, the stylus is longer than the cone, bears five to six prominent ridges, and exhibits a sharp apical protrusion [23,25,29]. In *Pontia*, the stylus is distinctly longer than the cone, bears four to five ridges of variable prominence, and terminates in a blunt apical protrusion [29]. These comparisons further support the usefulness of stylus morphology in comparative and taxonomic studies of Lepidoptera.

Across Lepidoptera, sensilla chaetica and basiconica are the most consistently present and widespread sensilla types, having been documented in Noctuidae [4,5,6], Pyralidae [9], Sphingidae [10], Hesperiidae [11], Nymphalidae [12,13], and Papilionidae [12]. However, several families also exhibit specialized sensillar subtypes. For example, *Helicoverpa armigera* (Noctuidae) possesses a distal subtype of sensilla chaetica and a curved-cone subtype of sensilla basiconica confined to the ventral proximal galea [4]. Sensilla styloconica, typically sparse and restricted to the distal proboscis in many taxa, have been described as forming dense, relatively uniform rows in Nymphalidae, resulting in a brush-like distal structure. Sensilla coeloconica, a distinct sensillum type, has also been reported in *Acherontia atropos* (Sphingidae) [10]. The increasing recognition of additional subtypes suggests a greater degree of functional diversification, potentially associated with dietary specialization.

The combination of proboscis regionalization and sensillum diversity in *P. edusa* may reflect functional adaptation to nectar feeding. The structurally robust Zone 1, characterized by abundant long sensilla chaetica and tightly interlocking legulae, may facilitate probing of narrow corolla tubes and provide mechanical feedback during insertion [19,31]. The grooved dorsal legulae in Zone 2 could enhance capillary forces or help regulate liquid flow, thereby potentially improving nectar uptake efficiency [18,39]. The flexible, legulae-free Zone 3 is likely to contribute to fine maneuvering of the distal tip, which may enable access to nectar concealed within complex floral structures [12]. The distribution of sensilla basiconica along the entire length of the galeae suggests a possible role in continuous evaluation of nectar quality, while internal sensilla basiconica may be involved in monitoring flow within the food canal [13,37]. The newly observed sc2 sensilla may enhance tactile sensitivity during coiling and could provide advantages when feeding on structurally complex or variable flowers. These functional interpretations remain tentative and require further experimental validation.

## 5. Conclusions

In conclusion, this study provides a systematic characterization of the zonal organization of the proboscis and the diversity of associated sensilla in adult *P. edusa*. Three morphologically distinct proboscis zones were clearly delineated, and sensilla chaetica subtype 2 (sc2) was identified for the first time on the ventral surface of the proboscis. These findings offer new morphological evidence for structural differentiation along the proboscis and expand current knowledge of sensillar diversity in Pieridae.

## Figures and Tables

**Table 1:** Morphological parameters of the proboscis and sensilla in *Pontia edusa*.

Structure	Length (μm)		*p*	*t*	Width/Basal Width (μm)		*p*	*t*
	Female (*n*)	Male (*n*)			Female (*n*)	Male (*n*)		
Proboscis	11,193.26 ± 931.10 (20)	10,706.71 ± 612.87 (24)	0.05	2	–	–	–	–
Zone 1	10,895.60 ± 953.08 (13)	10,000.44 ± 749.85 (21)	0.01 **	3.05	119.08 ± 14.23 (15)	117.77 ± 9.09 (22)	0.76	0.31
Zone 2	583.53 ± 61.38 (13)	644.88 ± 64.38 (21)	0.01 **	−2.75	42.30 ± 2.94 (11)	44.12 ± 2.80 (12)	0.14	−1.52
Upper branch of dorsal legulae in Zone 1	25.18 ± 2.33 (7)	20.93 ± 2.08 (10)	0.001 **	3.94	9.41 ± 2.22 (7)	8.31 ± 1.17 (11)	0.26	1.21
Upper branch of dorsal legulae in Zone 2	44.27 ± 5.38 (5)	36.25 ± 4.11 (5)	0.03 *	2.65	22.44 ± 1.52 (5)	17.41 ± 1.44 (5)	<0.001 **	5.36
Sensillum chaeticum 1	9.49 ± 1.81 (16)	9.58 ± 1.44 (20)	0.86	−0.17	1.48 ± 0.13 (16)	1.61 ± 0.22 (20)	0.04 *	−2.20
Sensillum chaeticum 2	19.83 ± 3.14 (3)	16.37 ± 2.07 (4)	0.14	1.78	1.96 ± 0.23 (3)	1.83 ± 0.34 (4)	0.59	0.58
Sensillum basiconicum 1	3.77 ± 0.49 (8)	3.83 ± 0.43 (9)	0.79	−0.27	1.48 ± 0.17 (8)	1.69 ± 0.21 (9)	0.04 *	−2.21
Sensillum basiconicum 2	1.65 ± 0.21 (13)	1.62 ± 0.18 (17)	0.68	0.42	0.99 ± 0.13 (13)	1.05 ± 0.17 (17)	0.31	−1.04
Sensillum basiconicum 3	9.78 ± 1.25 (5)	9.79 ± 1.72 (7)	0.99	−0.01	1.64 ± 0.21 (5)	1.67 ± 0.23 (7)	0.81	−0.25
Sensilla styloconica	10.72 ± 0.89 (9)	9.94 ± 0.76 (12)	0.04 *	2.15	6.36 ± 0.33 (9)	6.05 ± 0.33 (12)	0.05 *	2.12

Data of the proboscis and associated sensilla are presented as mean ± SD (*n*); *n*, sample size; * *p* < 0.05 and ** *p* < 0.01 in the independent samples *t*-test; –, unmeasured.

## Data Availability

The original contributions presented in this study are included in the article. Further inquiries can be directed to the corresponding author.
